# Structure-Based Drug Design for Cytochrome P450 Family 1 Inhibitors

**DOI:** 10.1155/2018/3924608

**Published:** 2018-07-25

**Authors:** Zbigniew Dutkiewicz, Renata Mikstacka

**Affiliations:** ^1^Department of Chemical Technology of Drugs, Poznań University of Medical Sciences, Grunwaldzka 6, 60-780 Poznań, Poland; ^2^Department of Inorganic and Analytical Chemistry, Ludwik Rydygier Collegium Medicum, Nicolaus Copernicus University in Toruń, Dr A. Jurasza 2, 85-089 Bydgoszcz, Poland

## Abstract

Cytochromes P450 are a class of metalloproteins which are responsible for electron transfer in a wide spectrum of reactions including metabolic biotransformation of endogenous and exogenous substrates. The superfamily of cytochromes P450 consists of families and subfamilies which are characterized by a specific structure and substrate specificity. Cytochromes P450 family 1 (CYP1s) play a distinctive role in the metabolism of drugs and chemical procarcinogens. In recent decades, these hemoproteins have been intensively studied with the use of computational methods which have been recently developed remarkably to be used in the process of drug design by the virtual screening of compounds in order to find agents with desired properties. Moreover, the molecular modeling of proteins and ligand docking to their active sites provide an insight into the mechanism of enzyme action and enable us to predict the sites of drug metabolism. The review presents the current status of knowledge about the use of the computational approach in studies of ligand-enzyme interactions for CYP1s. Research on the metabolism of substrates and inhibitors of CYP1s and on the selectivity of their action is particularly valuable from the viewpoint of cancer chemoprevention, chemotherapy, and drug-drug interactions.

## 1. Introduction

In the past decade, an interest in the use of computational methods in preclinical drug discovery has been continuously growing. Structure-based drug design (SBDD) became possible due to the availability of X-ray structures of receptors and the development of molecular modeling methods. The combined techniques employed in a drug discovery with respect to numerous receptors are still being improved and are becoming of better and better quality. There are thorough reviews of the actual possibilities of and prospects for using computational methods in drug design [1–5]. The present review is devoted to studies on cytochrome P450 family 1 (CYP1), an important family of enzymes responsible for drug metabolism and procarcinogen activation, with the use of molecular docking and molecular dynamics simulations. In some respects, family 1 of cytochromes P450 is exceptional. It comprises two isozymes, CYP1A1 and CYP1A2, of a great similarity and a third, more distinctive CYP1B1, which displays overlapping substrate specificity with the other members of the family. These three enzymes possessing relatively small binding cavities are valuable objects for comparative studies of enzymatic catalysis and ligand-enzyme interactions with the use of computational methods.

Cytochromes P450 (CYPs) are a superfamily of constitutive and inducible enzymes—hemoproteins—responsible for the oxidative metabolism of various xenobiotics and bioactive endogenous compounds. At present, 57 human genes of cytochromes P450 are known; they demonstrate a significant interindividual genetic variability [6]. On the basis of structural homology, CYPs may be assigned to the same family if they share not less than 40% of the amino acid sequence identity. Isoforms showing more than 55% sequence identity belong to the same subfamily. Family 1 (CYP1) comprises three isoforms: CYP1A1, CYP1A2, and CYP1B1. The amino acid sequence of CYP1A2 is 72% identical to that of CYP1A1, while CYP1B1 has lower amino acid sequence identity with both CYP1A1 (38%) and CYP1A2 (37%). Despite that, CYP1B1 is qualified as a CYP1 member on the basis of similar substrate specificity and the common induction of CYP1s by the aryl hydrocarbon receptor (AHR) [7].

CYP superfamily enzymes contain a heme prosthetic group catalyzing oxidation reactions and N- and O-dealkylations of substrates. CYPs are diversified in respect to substrate specificity and inhibitor susceptibility. Family 1 of these enzymes/CYP1s is responsible for the phase I metabolism of endogenous and exogenous substrates. CYP1s participate in the oxidative metabolism of endogenous substances, such as bile acids, steroid hormones, and lipids, exogenous compounds, and numerous pharmaceuticals and compounds derived from environmental pollution. CYP1s metabolize potential carcinogens: aryl hydrocarbons, aromatic amines, heterocyclic aromatic amines, and heterocyclic amines. CYP1s play a pivotal role in procarcinogen activation catalyzing metabolism of 66% of potential carcinogens [8]. The biotransformation of procarcinogens leads to the formation of mutagenic compounds, which form adducts with nucleic bases, responsible for the initiation of carcinogenesis.

CYP1B1 plays a particularly important role in pathogenesis of hormone-induced cancers, being responsible for metabolism of 17-alpha-estradiol (E2) to highly mutagenic and carcinogenic 4-hydroxy-E2 [9]. 4-Hydroxy-E2 and other products of E2 metabolism—quinones and semiquinones—exhibit genotoxic activity by forming adducts with nucleic acids [10]. Recently, CYP1B1's role in cancer progression and metastasis was reported [11]. CYP1A1 and CYP1B1 are targets of anticancer agents because of their overexpression in tumor cells compared to their normal counterparts [12, 13]. They may be used as a marker/tumor antigen in therapeutic strategies [14]. Overexpressed CYP1A1 or CYP1B1 in target tissues may play a double-sided role: they may activate prodrugs to their therapeutic forms, and on the contrary, they metabolize chemotherapeutics to their inactive forms. Inhibitors of CYP1B1 activity are used in mechanistic studies of drug metabolism [15].

Multidrug resistance caused by an efficient metabolism of chemotherapeutics catalyzed by CYP1A1 and CYP1B1 is a crucial problem in cancer chemotherapy. Moreover, inter- and intraspecies variability of CYP structures results in unique profiles of enzyme activities, which influence the therapeutic action of drugs metabolized by CYPs. The modeling of unique CYP structures may help to explain individual variation in response to drugs. To stop the inactivation of chemotherapeutics and to avoid drug resistance, the use of CYP1s inhibitors is proposed [9, 14–16].

The expression of CYP1 isoenzymes is tissue specific [17]. CYP1A1 is an inducible enzyme that occurs in the lungs and the trachea. Its induction is dependent on the presence of pollutants in the environment. CYP1A2 is a hepatic constitutive form of the enzyme that is responsible for drug metabolism in the liver. CYP1B1 occurs in the majority of extrahepatic tissues. A high level of CYP1B1 is observed in the bone marrow, kidney, spleen, thyroid gland, and reproductive tissues such as the uterus or prostate and mammary glands [17].

A focus on CYPs, in particular on CYP1s, arises from the role they play in the activation of procarcinogens. CYPs involved in the initiation of carcinogenesis have become the targets for anticancer strategy [18, 19]. One of the prophylactic actions is chemoprevention, which was defined as a prevention, inhibition, or reversal of carcinogenesis with the use of compounds of natural origin, their derivatives, or synthetic compounds [20]. The inhibition of CYP1A1 and CYP1B1 activities by natural compounds present in human diet constitutes one of the chemopreventive strategies [21].

In the past, the interactions between cytochromes P450 (CYPs) and their ligands have been investigated on the basis of the homology models of cytochrome P450 isozymes [22–24]. The homology models of CYPs had been used until the crystallographic structures of CYP1A1, CYP1A2, and CYP1B1 were determined and described [25–27]. Now, the crystal structures of CYP1s are available in the Protein Data Bank (PDB, http://www.rcsb.org/pdb/home/home.do), which greatly facilitates the progress of hit identifications.

**Figure 1:**
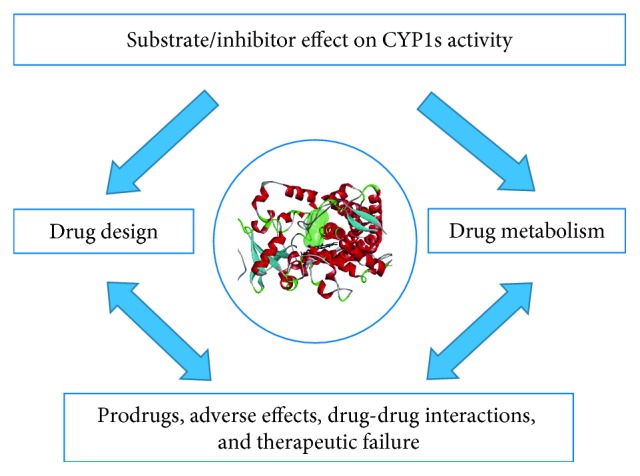
Relevance of CYP1 structure-activity relationship studies.

The computational approach in structure-activity relationship studies is conducted with the aim of determining the site of metabolism of drugs and prodrugs and explaining the mechanism of therapeutic failure, prodrug toxicity, and adverse effects (Figure 1). In computational-aided drug design at the “hit-to-lead” stage, two different approaches are used: the structure-based (or receptor-based) drug design (SBDD) and the ligand-based drug design (LBDD) in relation to the known structure of a receptor or a ligand.

This review summarizes in silico studies and the studies that combine investigations *in vitro* with a computational approach to CYP family 1 enzymes as targets for the inhibitory activity of ligands. In our review, we focused on the SBDD concerning CYP1 family enzymes. We surveyed the studies of interactions of lead compounds with CYP1A1, CYP1A2, and CYP1B1. We also discussed the studies focused on the search of compounds: substrates and inhibitors, which display selective and potent molecular interactions with individual isozymes.

## 2. Structures of CYP1 Family Members

### 2.1. CYP1A1

Before the structure of human CYP1A1 was determined, most reports concerning CYP1A1 were performed on homology models (Table 1). In the1990s, homology models were based on crystal structures of bacterial enzymes. The first reports involved the CYP1A1 model based on the structure of bacterial CYP102 (CYP BM3) [22]. The disadvantage of this model was a low sequence homology between bacterial and eukaryotic CYPs. A better homology was achieved for the CYP1A1 constructed by Szklarz and Paulsen [28] with the use of mammalian CYP2C5, the first crystal structure of a microsomal CYP 2C5 from a rabbit [84]. Since 2007, the crystal CYP1A2 structure has been available, and the CYP1A1 homology model based on this template was constructed, achieving a better stereochemical quality [34].

**Figure 2:**
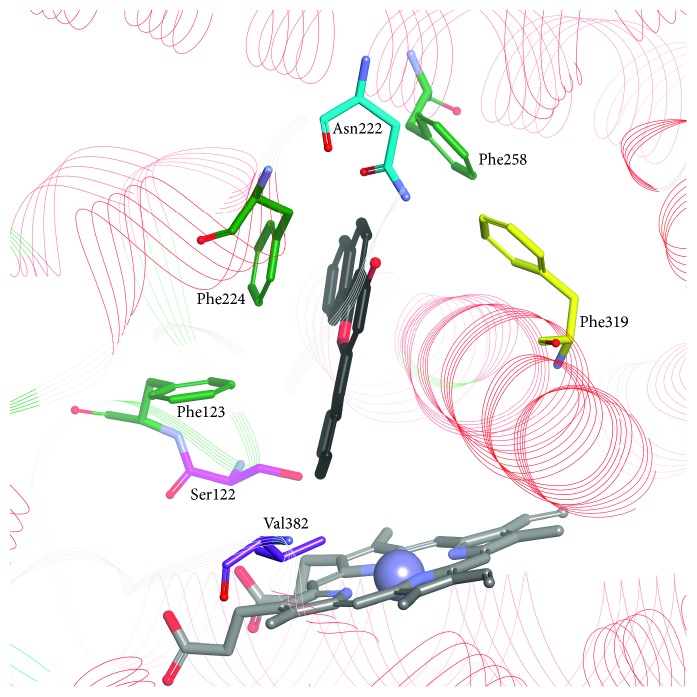
ANF bound in the active site of CYP1A1 (PDB ID: 4I8V). ANF: black carbon atoms; conserved phenylalanines 123, 224, and 258: light green; selected nonconserved residues: Ser122, Asn222, Phe319, and Val382.

The crystal 2.6 Å structure of human CYP1A1 (PDB: 4I8V; Figure 2) in complex with the inhibitor *α*-naphthoflavone (ANF) was determined by Walsh and coworkers in 2013 as the last of the three members of the CYP1 family [25]. The overall CYP1A1 structure displays the typical cytochrome P450 fold with canonical helices A–L and short F′ and G′ helices thought to be buried in the membrane and probably involved in enabling the access of hydrophobic ligands to the active site. A characteristic five-residue break in the middle of the F helix and lack of one of the four canonical *β* sheets that occur in human P450 enzymes were also found in the CYP1A1 crystal structure [25].

Recently, the CYP1A1 structure was used in studies of interactions of the enzyme with a great number of CYP1A1 substrates and inhibitors summarized in Table 1, including phytochemicals, for example, dietary flavonoids [32, 41, 47]; drugs, for example, melatonin, debrisoquine, theophylline, clozapine, and carvedilol [57]; environmental pollutants, for example, aromatic hydrocarbons and their derivatives [22, 28, 35, 37, 45, 55]; and natural and synthetic derivatives of *trans*-stilbene [25, 46, 63].

The CYP1A1 crystal structure was harnessed to identify new leads exhibiting CYP1A1-mediated anticancer activity [62]. The authors generated and validated a ligand-based pharmacophore model using a series of known anticancer compounds acting via CYP1A1. Selected compounds were subsequently subjected to pharmacokinetic screening, MetaSite screening, a molecular docking study, and a PAINS (pan-assay interference compounds) filter to refine the retrieved hits. Nine compounds capable of generating reactive metabolites and good interactions with CYP1A1 were selected for further studies *in vitro*. Two compounds showing a potent activity against the MDA-MB-435 cell line with IC_50_ < 0.1 *µ*M and low toxicity to normal cells were selected. These compounds were metabolized by CYP1A1 to the *N*-hydroxylated products that are potential genotoxic agents and may be responsible for a toxic effect of parent compounds. A molecular dynamics simulation analysis was used to visualize the orientation of hit molecules in a CYP1A1 binding site cavity, which promotes bioactive metabolite formation by N-hydroxylation [62].

### 2.2. CYP1A2

**Figure 3:**
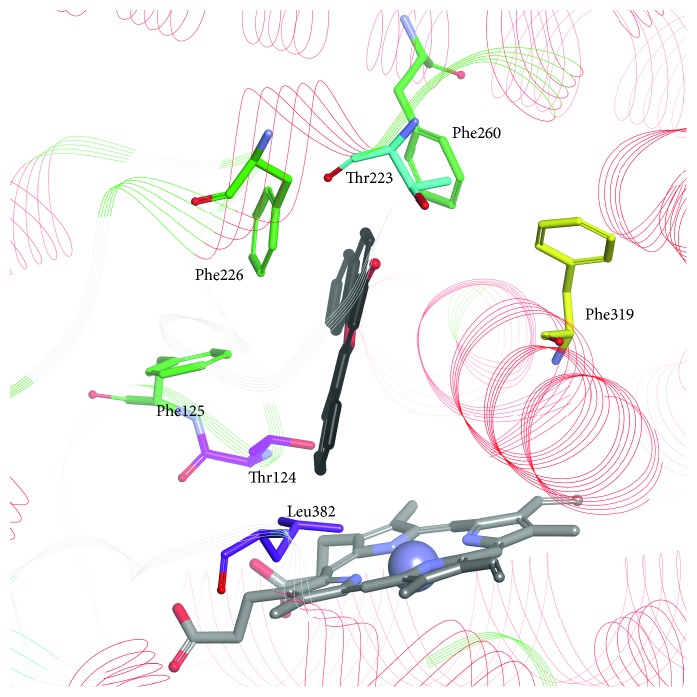
ANF bound in the active site of CYP1A2 (PDB ID: 2HI4). ANF: black carbon atoms; conserved phenylalanines 125, 226, and 260: light green; selected nonconserved residues: Thr124, Thr223, Phe319, and Leu382.

The crystal structure of CYP1A2 (PDB: 2HI4; Figure 3) was published in 2007, becoming a template for other members of the CYP1 subfamily [26]. This structure had been employed in the homology modeling of CYP1A1 and CYP1B1 until their crystal structures were determined. Site-directed mutagenesis and homology modeling studies of Wang and Zhou [85] revealed a series of residues in the substrate recognition sites (SRSs) of CYP1A2 (Arg108, Thr124, Glu225, Phe226, Lys250, Arg251, Lys253, Asn312, Glu318, Thr319, Asp320, Thr321, Vall322, Leu382, Thr385, and Ile386), which have been shown to play important roles in ligand-enzyme binding.

In the studies of flexibility of human cytochrome P450 enzymes (CYP1A2, CYP2A6, CYP2C9, 2D6, and CYP3A4) with molecular dynamics in combination with UV/Vis and resonance Raman spectroscopy, the active site of CYP1A2 was described as small and rigid in contrast to CYP3A4, which displayed a greater flexibility and the highest substrate promiscuity [74].

The narrow and flat binding pocket of CYP1A2 determines the substrate specificity of the enzyme. Phenacetin, ANF, furafylline, caffeine, and 7-methoxyresorufin serve as standard CYP1A2 inhibitors. Drug metabolism prediction with the use of docking, molecular dynamics, and quantum chemical methods was a good option to screen a library for potential inhibitors and drug-drug interactions [69, 86]. An efficient model for in silico screening was developed to identify CYP1A2 inhibitors in databases of herbal ingredients [73]. A rationale for these studies was herb-drug interactions. First, a pharmacophore model was constructed and validated. Then, the best pharmacophore model was chosen for a virtual screening of 989 herbal compounds. The hits (147 herbal compounds) were investigated through molecular docking and tested *in vitro*. Finally, 5 inhibitors of 18 candidate compounds were found to inhibit CYP1A2 activity. Molecular dynamics simulations provided an insight into the role of molecules of water in the enzyme active site. ANF forms the hydrogen bond with a water molecule, but during the simulations, different water molecules interact with ANF at different points of time [70].

Recently, more than 200 ns MD simulations were performed to investigate the role of water molecules in the active site of CYP1A2 complexed with 7-ethoxyresorufin and ANF [87]. Docking studies followed by MD simulations revealed that water molecules have an effect on hydrogen bond networks formed in the enzyme active site influencing the interactions of the substrate with amino acids in the enzyme active site. It appeared that water molecules were necessary for 7-ethoxyresorufin recognition, while for ligand recognition (ANF), water molecules were not required. The last conclusion is not consistent with the fact that, in the crystal structure of the CYP1A2 binding site (PDB: 2HI4), a water molecule is present. It is likely that the CYP1A2-ANF-WAT complex, with the crystal water molecule, did not reach equilibrium even during a longer (400 ns) simulation, which may be indicated by large fluctuations in RMSD for the ANF molecule, and the complex is in an intermediate state, between equilibrium and crystal structure.

### 2.3. CYP1B1

**Figure 4:**
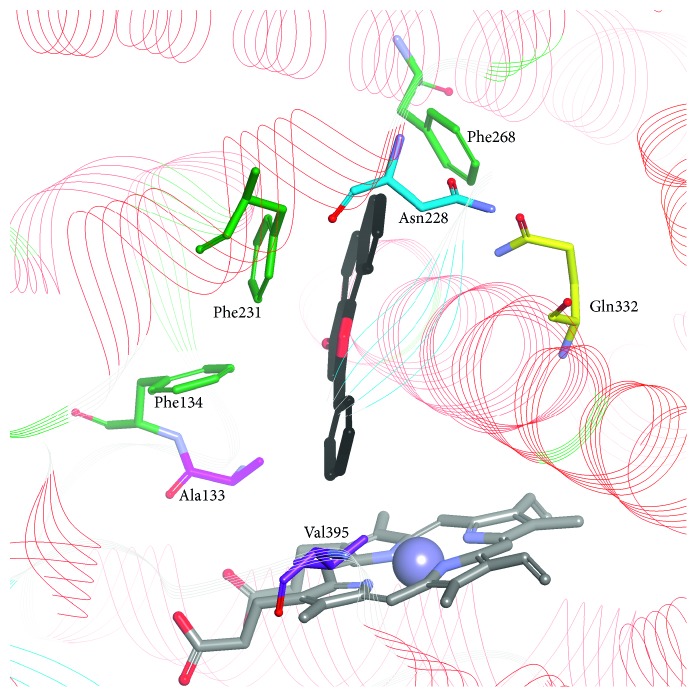
ANF bound in the active site of CYP1B1 (PDB ID: 3PM0). ANF: black carbon atoms; conserved phenylalanines 134, 231, and 268: light green; selected nonconserved residues: Ala133, Asn228, Gln332, and Val395.

The crystallographic structure of CYP1B1 (PDB: 3PM0; Figure 4) was determined by Wang and coworkers [27] with ANF as a ligand bound in the active site cavity. Like the CYP1A subfamily, CYP1B1 has a narrow active site. However, the sequence divergence causes a different orientation of ANF in CYP1B1, CYP1A1, and CYP1A2. Amino acids that line the edges of the cavities modify the substrate and inhibitor binding to CYP1B1 and other CYPs. In the characteristic distortion of the helix F of CYP1B1 and CYP1A2, *π*-*π* stacking interactions occur with Phe231 and Phe226, respectively. The amino acid residues Val395 and Ala133 determine the cavity shape in the vicinity of the heme. Val395 plays the role of Val382 in CYP1A1.

### 2.4. A Comparison of CYP1 Structures

A comparison of CYP1 family enzyme structures points to similarities and differences among the three active sites that determine their varied substrate specificities. The structures of all members of the CYP1 family were determined as complexes with ANF, making possible the comparison of ANF interactions in the active site cavities. ANF bound in the CYP1 cavities occupies the same plane adjacent to the heme and opposite to the I helix in each enzyme. The orientations of ANF in CYP1A1 and CYP1A2 are similar, whereas in CYP1B1, ANF is flipped by 180° about the long axis of the ligand.

The most meaningful differences in the structure were listed and analyzed by Walsh et al. [25]. The residue at position 382 is known to be important in determining the functional differences between CYP1A1 and CYP1A2; mutations at this position have a significant impact on the catalytic efficiency of enzymes; the smaller 382 residue facilitates ligand placement in the enzyme cavity. In CYP1A1, the only interaction formed by ANF is *π*-*π* stacking with Phe224 that is situated on the opposite side of I helix. In the CYP1A2 active site, there is a water molecule that forms hydrogen bonds with carbonyl groups of ANF and Gly316 [25]. A five-residue break in the CYP1A1 F helix has an effect on ligand binding, increasing the flexibility of the active site. The volumes of the active sites of CYP1A1, CYP1A2, and CYP1B1, which are 524, 375, and 398 Å^3^, respectively [25–27], and crucial amino acid residues in the enzyme cavity determine the shape of the best-fitted ligands. A triangle with the side length of 9.3, 8.7, and 7.2 Å was proposed as a contour of selective CYP1A2 inhibitors [53]. Moreover, CYP1A1 side chains of amino acid residues lining the active site are smaller in comparison to the corresponding residues in CYP1A2. For example, amino acid residues in the vicinity of the heme, Val382 in CYP1A1 and Val395 in CYP1B1, are replaced by the branched Leu382 in CYP1A2 narrowing the CYP1A2 cavity, which results in a lesser affinity of polymethoxystilbenes to CYP1A2 in comparison to CYP1A1 [63].

To investigate the active sites of CYP1s, a series of potential inhibitors were synthesized and tested for their inhibitory activity. Synthesizing two series of chemical probes: *α*-naphthoflavone-like and *β*-naphthoflavone-like pyranoflavones, Liu et al. created ligand models of CYP1A1 and CYP1A2. The molecular surface images were generated in UCSF Chimera 1.6.2. (UCSF San Francisco, CA) after energy minimization using the conjugate gradient method with the CHARMM force field. The authors concluded that CYP1A1 has a narrow and long cavity, 15.8 Å in length and 4.6 Å in width. The CYP1A2 cavity can accommodate a triangular molecule, showing a planar heart-like structure with a 9.1 Å long side and a 7.0 Å short side [53]. According to that suggestion, a series of 14 flavone and coumarin derivatives exhibiting a triangular planar shape were designed with the use of the computer-assisted alignment assay. Most of the tested compounds (13 out of 14) appeared to be selective CYP1A2 inhibitors. 4-Trifluoromethyl-7,8-pyranocoumarin and 7,8-furanoflavone were found to be the most effective CYP1A2 inhibitors with *K*_*i*_ at a submicromolar level [88].

In conclusion, the detailed topology of the CYP1A1 active site is more similar to the CYP1B1 cavity than to the cavity of CYP1A2 [25]. The closer similarity between the CYP1A1 active site and CYP1B1 compared with the CYP1A2 active site may influence the substrate profiles of these enzymes, which are more similar for CYP1A1 and CYP1B1 than for CYP1A1 and CYP1A2.

## 3. Mutations

The molecular modeling of enzymes differing in a selected amino acid sequence provides a rationale for substrate specificity. Studies of the relationship between an amino acid sequence and the functionality of CYP1s were possible, thanks to mutagenesis methods. To reduce the number of mutants to be constructed, the residues for replacement should be located in the active site of the enzyme and must be different from the corresponding residues in the enzyme which is being compared. Based on the knowledge of which active site residues are different between CYP1A1 and CYP1A2, the effect of reciprocal mutations on substrate specificity was examined [31]. The residue replacement in the substrate recognition site (SRS) reduced 7-methoxyresorufin (7-MR) and 7-ethoxyresorufin (7-ER) *O*-dealkylase activities, except for the CYP1A1 S122T mutation which increased both activities. The results confirmed the importance of SRSs for enzyme-substrate interaction, proposed earlier by Gotoh [89].

Functional alterations as a result of genetic polymorphism may influence the therapeutic response of many drugs changing their efficacy and toxicity. CYP1A2 participates in the metabolism of 9% of all medicines [6]. The CYP Allele Nomenclature Committee (http://www.cypalleles.ki.se/cyp1a2.htm) has recognized 40 CYP1A2 variant alleles. A functional characterization of 20 allelic variants of CYP1A2 was performed with two substrates: phenacetin and 7-ethoxyresorufin [90]. Four of the studied alleles, which exhibited the substitutions critical to the enzymatic function (located in: SRS, the heme-binding region, the aromatic region, and the proline-rich region), showed reduced activity toward both substrates. However, the substitution Arg377Gln might cause a change in hydrogen bonds to alternative ones with other amino acids, which resulted in the loss of enzyme activity or decreased holoprotein level. Two variants with the substitutions Thr438Ile and Asp436Asn showed a significantly higher activity toward phenacetin than the wild-type enzyme. Interestingly, the amino acid residues Thr438 and Asp436 are not located in the substrate binding site. They are situated on the surface of CYP1A2 and may influence the interaction of the enzyme with cytochrome b5 [90].

Zhang et al. [78] applied molecular dynamics simulations and structural analyses to elucidate mechanisms of mutation-induced allostery in CYP1A2. They explored the effects of a peripheral mutation, F186L, at ∼26 Å away from the enzyme active site on the enzyme catalytic activity. For these mutations, they found a change in protein flexibility and a collective protein motion that caused the main substrate access channel to be mostly closed. Dynamics simulations were used to explain the mechanism of a changed binding of 7-ethoxyresorufin in the catalytic pocket of the F186L mutant enzyme. Ma et al. demonstrated an impact of F186L mutation on the function of CYP1A2 [91]. Despite the fact that the mutation Phe186Leu is located on the surface, a series of changes in the catalytic pocket were observed. Phe186Leu mutation enhanced the binding affinity but lowered the O-deethylation velocity of 7-ethoxyresorufin. It was suggested that channel 2c, which is the main active channel in CYPs [92], is closed in the mutant CYP1A2 enzyme as a result of B'helix/B-C loop stabilization.

Allelic CYP1B1 variants were constructed in studies of the substrate metabolism catalyzed by CYP1B1 [82, 93, 94]. Mutant forms of CYP1B1 have been discovered in the childhood disease primary congenital glaucoma (PCG). Homology-modeled structures of wild-type and disease-associated mutant forms were constructed on the basis of human CYP2C9. In the mutant form of CYP1B1, changes in the geometry of the substrate binding region and the position of the heme were observed. Using molecular dynamics simulations, altered interactions of estradiol with the disease mutant of CYP1B1 in comparison with the wild type of enzyme were demonstrated [83].

More recently, the structures of eight mutants differing only in one residue were generated from the crystal structure of CYP1A2. Mutation of only one amino acid changed the enzyme static structure even in distant regions of the protein and influenced the flexibility of the whole protein and influenced the catalytic activity of the enzyme by changing the conformation of a ligand-enzyme complex [81]. Significant changes in the dynamic properties of CYP1A2 were observed when long-time MD simulations (100 ns or longer) were used.

## 4. The Molecular Docking of Ligands

Molecular docking is a computational approach which predicts the orientation of a ligand (*pose*) in complex with a protein target and assesses its binding affinity using scoring functions.

Structure-based drug design is performed in order to identify bioactive compounds in the compound pool found in high-throughput virtual screening (HTVS) based on the information from the protein structure. In structure-activity relationship studies, molecular docking helps to elucidate the bioactivity of lead compounds identified at the “hit-to-lead” stage on the basis of a ligand-target interaction analysis.

A computational approach predicts the orientation of ligands in complex with a protein target using scoring functions (specific algorithms). Structure-based virtual screening is a quick and more economical method of lead identification than experimental screening. In research, popular open-source docking software and more advanced commercial packages are used. Nonetheless, in the opinion of many authors, they still need to be improved to obtain a better pose prediction capability. The main factors limiting the accuracy of docking results are protein flexibility and solvation. The affinity of a ligand to a protein target is characterized by scoring functions which represent a relative binding free energy based on protein-ligand interactions. Scoring functions do not consider the contribution of thermodynamic effects on binding free energy like solvation, long-range interactions, and conformational changes. Protein-ligand docking methods are widely used at different stages of the drug design process. They are employed at the beginning for the virtual screening (VS) of large ligands' databases and at the lead optimization stage.

The scoring function is used by the searching algorithm to identify the best pose of a particular ligand, the most energetically favoured orientation inside the active site. It also estimates the binding affinity of a ligand. This allows us to rank ligands in virtual screening, where large and chemically diverse databases should be docked very effectively; so here, the speed of docking is more important than its accuracy [5]. However, in lead optimization, researchers are interested in obtaining docking results that are as accurate as possible for a small set of ligands, which are often structurally related. Besides assessing binding affinities to the macromolecular target for close analogues in lead optimization, docking can also be used for predicting off-target binding to related proteins and to cytochromes as drug-metabolizing enzymes [5].

The role of a docking algorithm is to generate ligand poses inside the binding site. The scoring function should correctly recognize the bioactive orientation and assign a sufficiently high score to it, allowing us to discriminate binders from nonbinders in terms of calculated binding affinity [5]. Scoring functions are classified as force field-based, empirical, and knowledge-based [95]. Force field-based functions account for electrostatic and van der Waals interactions in protein-ligand complexes using force field parameters. In empirical scoring, functions are terms describing specific ligand-protein interactions, for example, hydrogen bonds, ionic interactions, or hydrophobic effects. Another class of scoring functions, knowledge-based, was derived from a statistical analysis of the crystal structures of ligand-protein complexes. It does not use information about experimental activity but analyzes the distribution of ligand-protein atom pairs giving pairwise potentials [95].

All scoring functions have some limitations. They perform much better in identifying correct poses of individual ligands than in ranking ligands according to their activity for respective targets. Difficulty in differentiating between nano- and micromolar compounds limits the reliability of docking [5]. To overcome this issue, more than one scoring function can be employed in assessing binding affinity. Consensus scoring combines the results of several scoring functions; this approach is in some cases more successful in predicting activity than a single function [95].

Some specific interactions, for example, cation-pi, CH-pi, or weak hydrogen bonds, are not captured by commonly used scoring functions. Also, many simplifications, such as treating solvation effects and contributions of entropy to the binding energy, result in a poor ranking of compounds in VS [95]. Therefore, more advanced and computationally demanding methods for rescoring docked poses are applied. For this purpose, physics-based methods and simulations based on force fields and implicit solvent models are employed. Among them, commonly used approaches are the molecular mechanics-Poisson–Boltzmann surface area (MM-PBSA) and the computationally less demanding molecular mechanics-generalized Born surface area (MM-GBSA).

Many docking algorithms treat the receptor as conformationally rigid, which is a severe approximation influencing the final results. In fact, upon binding to a protein, ligands often induce changes in its conformation [5, 96]. The flexibility of a protein can be included in the macromolecular model in several ways. The simplest one is using “soft” receptors (*soft docking*) with decreased energy penalties for steric clashes between the ligand atom and the receptor. Other docking methods accounting for protein flexibility are a docking using side-chain flexibility, in which side-chain rotations of residues in the binding site are allowed, a docking using an ensemble of receptor structures (experimental or simulated), and on-the-fly docking, where protein conformations are generated “on the fly” during docking by exploring the protein's degrees of freedom [5, 97].

There are many examples of ligand-target interactions via water molecules (e.g., hydrogen bonding), so neglecting water molecules could be an additional source of errors in docking. Usually, before docking, water molecules are removed from the binding site, but there are also other options, such as keeping or displacing water molecules which are placed in the binding site or are important for the binding of ligands [5, 95].

Existing scoring functions are not perfect in ranking compounds in virtual screening and estimating absolute binding affinities in lead optimization. Also, receptor flexibility needs to be taken into account during docking experiments. Therefore, docking methods are still under development regarding aspects such as receptor flexibility, structural water or the solvation, and entropic effect [98].

Docking can predict a plausible orientation and conformation of ligands inside the binding site of the receptor, although this method gives only a static picture of ligand-receptor interactions. A deeper insight into the time-dependent properties of ligand-protein complexes could be obtained with the use of molecular dynamics (MD) simulations. In molecular dynamics simulations, solvent molecules are included explicitly or with the use of implicit solvent models. MD, an invaluable tool in SBDD, has many applications. Before docking, MD simulations could be used to give an ensemble of protein structures, but for postdocking complexes, this method allows for a computational testing of its stability and is often used to rescore docked ligands because of the improvement in the mutual fit and optimization of interactions that occur during the simulation. Calculations of binding free energy (Δ*G*_bind_) could be made using different methods, such as thermodynamic integration (TI), free energy perturbation (FEP), linear interaction energy (LIE), and the aforementioned MM-PBSA or MM-GBSA approaches [99]. There are many examples of successful applications of MD in the characterization of ligand-macromolecular target complexes [4, 99].

Protein-ligand binding energy should be determined as a nonadditive effect, which depends on the chemical environment and protein-ligand cooperative dynamic processes. Molecular dynamics simulation improves predictions of binding free energy by considering the time-dependent behaviour of the macromolecular system in response to changes in its molecular environment. However, docking results are not always consistent with MD simulation (different poses observed by docking and MD; a ligand does not form a long-lasting complex). It also happens that docking results are not proved by the biochemical assay *in vitro*, and vice versa; compounds with high bioactivities are shown to have a poor docking score. Many scientists point to the limitations of docking procedures [2, 69]. Table 1 presents a survey of studies devoted to computer-aided analysis of interactions of CYP1 enzymes with their ligands.

## 5. Substrates and Inhibitors of CYP1s

A classification of inhibitors and noninhibitors of CYPs is particularly important in relation to drug design and the prediction of drug-drug interactions. CYP1A2 is responsible for the biotransformation of ∼5% of currently used drugs. Screening a set of compounds from a database in search of CYP1A2 ligands seems to be more efficient than an experimental determination of catalytic activities of a series of compounds. However, the use of scoring functions did not always give satisfactory results. Better results were achieved with recently developed nonlinear machine learning methods. Seven thousand test compounds from a database were analyzed as CYP1A2 inhibitors. The accuracy of the developed method for the prediction of inhibitory activity was estimated at 73–76% [86], while the decision tree model based on Lipinski's rule of five classified 67% of the test compounds correctly. Binding free energies of structurally diverse CYP1A2 substrates and inhibitors were predicted with the use of the linear interaction energy (LIE) method. For 10 compounds (from the set of 13 test ligands), the difference between the calculated and experimental binding free energies was smaller than 4.0 kJ/mol [70]. CYP1A2 ligands were identified from a large compound library (16,338 compounds) with the use of two approaches: structure-based and ligand-based virtual screening. As compared to the ligand-based method, the structure-based method identified more inhibitors which were more potent as well [100].

In this review, we present studies on specific interactions of substrates/inhibitors with CYP1 isozymes, which allowed for an analysis of the relationship between the structure of the tested compounds and their inhibitory activities. The studies on ligand-CYP1 enzyme interactions with the use of computational methods are summarized in Table 1. There are some groups of compounds that are particularly interesting, and many reports devoted to their interactions with CYP1s are discussed below. These are endogenic substrates, alkoxyresorufins, polycyclic aromatic hydrocarbons, and compounds that are supposed to play a role in cancer chemoprevention: natural flavonoids and *trans*-stilbene derivatives. The bioactivity of natural chemopreventive agents inspired researchers to synthesize their derivatives in order to study the structure-activity relationship and to obtain more active and efficient chemopreventive agents.

### 5.1. Endogenic Substrates

17-*β*-Estradiol (E2) is metabolized by CYP1s to 2-hydroxy, 4-hydroxy, or 16-hydroxy derivatives. The order of preference for *in vitro* 2-hydroxylation by CYP1 isoforms was CYP1A2 > CYP1A1 > CYP1B1; for 4-hydroxylation, it was CYP1B1 > CYP1A2 > CYP1A1; and for 16-hydroxylation, CYP1A2 showed the highest preference followed by CYP1A1 and CYP1B1. In the mammary gland, CYP1A1 catalyzes predominantly 2-hydroxylation. 2-Hydroxyestradiol (2-OHE_2_) is further methylated by catechol-*O*-methyltransferase to produce 2-methoxyestradiol, which does not exhibit carcinogenic activity. On the contrary, it inhibits the proliferation of cancer cells. 4-Hydroxyestradiol (4-OHE_2_) is produced in a reaction catalyzed mainly by CYP1B1 [101, 102]. The product of 4-OHE_2_ oxidation (estradiol-3,4-quinone) forms quinone-DNA adducts and initiates carcinogenesis [103]. With the use of homology models of CYP1A1 and CYP1B1 based on the crystal structure of CYP1A2, Itoh et al. analyzed the structural causes of different sites of E2 metabolism [44]. The studies revealed one binding mode of E2 (18-methyl group up) to CYP1A1 and CYP1A2 and two binding modes of E2 (18-methyl group up and down) to CYP1B1. Thr124 and Phe260 of CYP1A2 and Ser122 and Phe258 of CYP1A1 were identified as causing steric hindrance with the B-ring of E2. Ala133 and Asn265 of CYP1B1 are critical residues influencing the interaction of E2 with the binding site. Conformations of E2 in enzyme cavities decided on the site of E2 metabolism leading to the hydroxylation preferentially at the position 2 in case of CYP1A1 and CYP1A2, and at the position 4 in CYP1B1 [44].

Fatty acids are an essential class of CYP endogenic substrates whose metabolites are supposed to play a physiological role in the cardiovascular system. With the use of molecular docking, regiospecificity of the metabolism of arachidonic acid (AA) and eicosapentaenoic acid (EPA) catalyzed by human recombinant CYP1A1 in the reconstituted enzymatic system was studied. Interestingly, AA was mainly metabolized to 19-hydroxyarachidonic acid by CYP1A1. With EPA as a substrate, CYP1A1-dependent epoxygenase activity leading to the regiospecific and stereoselective formation of 17(*R*),18(*S*)-epoxyeicosatetraenoic acid (68%) and 19-hydroxy-EPA (31%) was demonstrated [30]. The molecular docking of AA and EPA to the CYP1A1 active site revealed that fatty acids interact with the same amino acid residues as alkoxyresorufins and benzo(*a*)pyrene, although additional residues located in the access channel may interact with AA and EPA owing to their longer molecules. Conformations of fatty acids in the CYP1A1 binding site are stabilized at their carboxy ends by hydrogen bonds, while resorufin and benzo(*a*)pyrene are mainly stabilized by hydrophobic interactions [30]. The complexes of AA and EPA with CYP1A1 were further examined with MD simulations to obtain productive binding modes. The in silico site scoring of geometric criteria, angles and distances of the substrates to the ferryl oxygen, confirmed that steric factors play a key role in the regiospecificity of CYP1A1-mediated metabolism [33].

### 5.2. Alkoxyresorufins

Alkoxyresorufins are CYP1 substrates used in activity assays. Lewis and Lake [22] initiated the computational approach in the studies of substrate affinity to CYP1 binding sites. In the 1990s, homology models of CYP1A1 and CYP1A2 binding sites were generated from the bacterial CYP102 crystal structure via residue replacement and energy minimization procedures, and a series of known substrates and inhibitors of the CYP1A subfamily were docked interactively to the active sites [22]. The orientation of 7-MR and 7-ER in binding site cavities was determined with the aim of elucidating their substrate specificity; 7-MR is a specific substrate of CYP1A2, while 7-ER demonstrates a higher affinity to CYP1A1 over CYP1A2. The enzymes share 72% of the amino acid sequence identity; however, the differences in their structures seem to be sufficient to explain their specific affinity to the ligands. Critical changes in the CYP1A1 and CYP1A2 structures were found. In the I helix, the change from aspartate adjacent to Thr268 in CYP1A1 to glutamate in CYP1A2 gives rise to the steric restriction in the CYP1A2; as a result, there is no sufficient space for 7-ER in the binding site of CYP1A2. Moreover, in the F helix, which lies above the heme moiety, there are amino acid residues that are donors of hydrogen for the carbonyl group being located in resorufins on the opposite side of a molecule; in CYP1A1, the carbonyl group of 7-ER can form a hydrogen bond with Thr185, while 7-MR can form a hydrogen bond with Asp184 in CYP1A2 [22].

Szklarz and Paulsen [28] docked 7-MR and 7-ER manually to the CYP1A1 binding site (homology model generated from CYP2C5) in the orientations, leading to the formation of major products. The residues located within 5 Å were identified. Val382 was found as a key residue that stabilized 7-ER in the CYP1A1 binding site through van der Waals interactions [28]. This interaction did not occur in the case of 7-MR. Moreover, a higher activity of CYP1A1 toward 7-ER may be explained by interaction energy, which is significantly higher for 7-MR (lower absolute value). The effects of five key residues—Ser122, Asn221, Gly225, Leu312, and Val382 in CYP1A1, and Thr124, Thr223, Val227, Asn312, and Leu382 in CYP1A2—on the substrate specificity of enzymes were investigated [31]. Specificity changes were observed, but no single mutation that could confer the activity of one isoform onto another was found. As a continuation of studies, 26 possible multiple mutants of CYP1A2 were constructed and investigated with the molecular dynamics-based scoring method. In 7 mutants, the specificity shift from CYP1A2 to CYP1A1 was predicted. For 5 mutants, the prediction was confirmed by site-directed mutagenesis and biochemical assays [68].

When 7-ER was docked to CYP1A1 generated using CYP1A2 as a template, the amino acid residues found within a 3 Å radius from the substrate were Ser120, Ser12, Phe123, Phe224, Phe258, Tyr259, Asp313, Thr321, Val382, and Ile386. For this CYP1A1 structure, substrate inhibition kinetics was observed, probably due to a nonproductive orientation of 7-ER in the CYP1A1 binding site. Docking studies showed that the symmetrical molecule of 7-ER may be bound in a reverse orientation with the ethoxy group directed in the opposite side of the heme, which is energetically favourable in the CYP1A1 wild type and mutants [34].

### 5.3. Polycyclic Aromatic Hydrocarbons

Polycyclic aromatic hydrocarbons, present ubiquitously in the environment, are planar aromatic compounds produced mainly in combustion processes. Benzo(*a*)pyrene is a procarcinogen activated by CYPs to mutagenic products which form adducts with DNA. Its metabolism by CYP1s has been studied with the use of molecular docking since the 1990s [22]. The studies have been continued by Szklarz and collaborators [28, 33], who found a correlation between the numbers of docked orientations within 4 Å of the ferryl oxygen and experimentally determined metabolite ratios. The regiospecificity of B(*a*)P metabolism was demonstrated with a homology model based on the CYP 2C5 crystal structure of CYP1A1 [33] and with the use of multiple models of killifish, scup, rat, and human CYP1A1s [37]. In all the models analyzed, the 8,9-bond was more frequently close to ferryl oxygen than 7,8- or 9,10-positions. However, 8,9-epoxide production has never been observed owing to unfavourable formation energy. The formation of epoxides in the close vicinity of 8,9-position—7,8-epoxide or 9,10-epoxide—is supposed to be a result of a small reposition of a substrate molecule by vibration or rotation within the active site [37].

CYP1B1 inhibition by eleven polycyclic aromatic hydrocarbons (PAHs) and 14 acetylenic PAHs and biphenyls was studied. Five of the potent inhibitors with IC_50_ at the nanomolar level (benzo(*a*)pyrene, dibenzo[*aj*]acridine, 1-(1-propynyl)pyrene, 3-(1-propynyl)phenanthrene, and benzo[*j*]fluoranthene) were docked to the CYP1B1 and CYP1A2 cavities showing different binding modes for selected aromatic hydrocarbons [104].

An alternatively spliced variant of CYP1A1 having a deletion of exon 6 was discovered in human brain tissue [35]. The lack of B(*a*)P metabolism to genotoxic ultimate carcinogens by the exon 6 del CYP1A1 was elucidated by molecular docking studies. B(*a*)P docked to the wild CYP1A1 (being modeled with the CYP2C5 crystal structure) was situated in a way that made possible an oxidation reaction in the positions 7, 8, 9, and 10 of the aromatic ring. Two major clusters of orientations were found, out of the 6 observed for the B(*a*)P molecule docked to the CYP1A1 binding site: the first with 7, 8, 9, and 10 positions near the heme iron (72% of all 50 studied conformations) and the second with position 3 close to the heme (14% of conformations). Among 11 orientations found for B(*a*)P in the exon 6 del CYP1A1 active site, in two main orientations, C-3 was in a close proximity to the heme [99]. However, the 3-hydroxylated product of B(*a*)P metabolism is not considered as genotoxic. B(*a*)P was differentially orientated in the CYP1A2 and CYP1B1 binding sites; positions 7, 8, 9, and 10 of the aromatic scaffold were observed in proximity to the heme iron only in the CYP1A2 binding cavity [104].

A collection of 22 polycyclic aromatic hydrocarbons of increasing size were docked to wild-type and chimeric CYP1A enzymes. The QSAR analysis revealed that the size of the substrate influences its accessibility to the enzyme cavity via access channels. A visualization of CYP1A enzymes with the use of CAVER software showed two regions located close to or within the CYP access channels affecting differentially small and large polycyclic substrates [58].

Polychlorinated dibenzo-*p*-dioxins (PCDDs) and coplanar polychlorinated biphenyls (PCBs) are a class of aromatic hydrocarbons demonstrating high genotoxicity. The metabolism of dioxins and PCBs shows the species-based differences between humans and rats [55]. Human CYP1s metabolized efficiently low-chlorinated PCDDs, while 2,3,7,8-tetrachlorodibenzo-*p*-dioxin (TCDD) metabolites were not detected. Rat, but not human, CYP1A1 metabolized 3,3′,4,4′,5-pentachlorobiphenyl, the most toxic PCB, to two hydroxylated derivatives showing lower toxicity than the parent compound. Docking studies with the use of homology models of human and rat CYP1A1 indicated essential amino acids residues (Ala120 and Phe316) for 3,3′,4,4′,5-pentachlorobiphenyl metabolism. The differences in amino acid residues led to changes in the size and shape of the cavities; in the rat CYP1A1 cavity, 3,3′,4,4′,5-pentachlorobiphenyl was close enough to the heme to be metabolized [45].

Species-based differences were studied by the docking of B(*a*)P, 3,3′,4,4′-tetrachlorobiphenyl (TCB), and TCDD to multiple models of rat, human, killifish, and scup CYP1A1 [37]. Mutating interacting residues of killifish CYP1A1 to corresponding residues of human CYP1A1 led to TCB poses similar to those of human CYP1A1. A slower oxidation of TCDD in comparison to TCB by each species may be explained by structural constraints in the enzyme binding site. A slower metabolism of TCDD by human CYP1A1 than rat CYP1A1 resulted from the lower frequency of productive poses in human CYP1A1.

The molecular docking of 37 polycyclic aromatic hydrocarbons, corresponding diols, and heterocyclic hydrocarbons to homology models of CYP1A1 and CYP1B1 based on the crystal structure of CYP1A2 was performed with LigandFit and CDOCKER algorithms [50]. The analysis of CYP1A1 and CYP1B1 binding sites revealed their hydrophobic character due to hydrophobic residues, mainly the phenylalanines Phe123, 224, and 258 in CYP1A1 and Phe134, 231, and 268 in CYP1B1, which may interact through *π*-*π* stacking with aromatic ligands. However, potential hydrogen bond donor residues, Ser122, Asn221, Leu312, Asp313, Gly316, Ala317, and Asp320, found in the CYP1A1 binding site and corresponding residues in CYP1B1, Ala133, Asn228, Thr325, Asp326, Gly329, Ala330, and Asp333, stabilized the ligand molecules by hydrogen bonds. The amino acid residues which mainly interact with the ligands under study are located in the substrate recognition sites classified by Gotoh [89]. Interestingly, the CDOCKER docking procedure gave the best results for CYP1A1 linear statistical analysis, while LigandFit appeared to be a more suitable procedure for CYP1B1 [50].

### 5.4. Flavonoids

Flavonoids are a large class of natural bioactive compounds present in fruits and vegetables. Their role in cancer prevention is established in epidemiologic studies [21]. In experimental *in vitro* studies, flavonoids appeared to be potent inhibitors of CYP1s. A correlation was found between the inhibition of CYP1A1 and CYP1A2 activities by flavonoids differing in the position and number of hydroxyl groups and theoretical descriptors obtained from quantum mechanical calculations and molecular dynamics of the ligand-enzyme complex [32]. In this report, quercetin and kaempferol docked to the binding site of CYP1A2 were demonstrated, and amino acid residues responsible for ligand-enzyme interactions that may be useful in site-directed mutagenesis were found. Takemura et al. demonstrated a selective inhibition of CYP1B1 by flavonoids, particularly chrysoeriol and isorhamnetin. To explain their strong effect on CYP1B1, a molecular docking approach was employed [42]. For this purpose, they constructed three-dimensional structures of CYP1A1 and CYP1B1 by homology modeling, using the crystal structure of CYP1A2. The authors concluded that methoxyflavonoids—chrysoeriol and isorhamnetin—fit well into the active site of CYP1B1, while in active sites of CYP1A1 and CYP1A2, there occurred a steric collision between methoxy substituents and Ser-122 in CYP1A1 and Thr-124 in CYP1A2. The binding specificity of methoxyflavonoids is based on interactions between methoxy groups and specific CYP1 residues. Methoxyflavonoids possessing a 2-3 double bond in the C-ring, as selective inhibitors of CYP1B1, are supposed to be chemopreventive agents against CYP1B1-related carcinogenesis.

Oroxylin and wogonin are biologically active compounds occurring in the extract of roots of *Scutellaria baicalensis*, used in traditional oriental medicines [76]. Oroxylin and wogonin are inhibitors of CYP1A2 with IC_50_ values of 579 and 248 nM. With the use of molecular docking, molecular dynamics simulation, and MM-PBSA, the mechanism of the inhibitory action of flavonoids differing in the position of a hydroxyl group was analyzed. Calculated binding free energies of ANF (−23.5 kcal/mol), wogonin (−21.1 kcal/mol), and oroxylin (−19.8 kcal/mol) are significantly overestimated; however, they are in accordance with the order of experimentally determined inhibitory activities. The difference in the affinity of oroxylin and wogonin to the CYP1A2 active site was explained by molecular dynamics and molecular docking; for ANF and wogonin, noncovalent interactions (van der Waals and hydrophobic interactions) influenced the stability of their complexes with CYP1A2. In the CYP1A2-oroxylin complex, there occurred an energetically unfavourable repulsion between Thr118 and the methoxy group at position 6 in the oroxylin molecule. As a result, conformational changes in the side chain of Thr118 were observed, which caused the formation of a more open and larger binding site cavity of CYP1A2 and a weaker inhibitory activity of oroxylin. Moreover, the O7 atom of oroxylin formed a strong hydrogen bond with Asp313, as the O5 and O6 atoms formed two hydrogen-bonding interactions with a molecule of water. These interactions were not observed in complexes of CYPA2 with wogonin and ANF [76].

Chrysin (5,7-dihydroxyflavone), a natural, biologically active flavonoid extracted from plants and honey, exhibited an inhibitory activity toward CYP1A2 comparable to ANF (IC_50_ values of 54 nm versus 49 nM). With molecular docking and molecular dynamics simulations, the interactions in the enzyme binding site were estimated. The complex of chrysin with CYP1A2 was stabilized with van der Waals interactions, H-bond with Asp313, and stacking interactions with Phe226 [71]. The affinity of chrysin to CYP2C9 was significantly weaker because van der Waals interactions in the larger pocket of CYP2C9 were not as strong as in CYP1A2.

Based on the known inhibitory activity of compounds, more efficient inhibitors can be designed. Flavone derivatives with an acetylene group linked to the flavone backbone showed a comparable ANF inhibitory activity against CYP1A1. Moreover, mechanism-based inactivators of CYP1A1 were found. 4′-Ethynylflavone and 7-ethynylflavone irreversibly inactivated half of the CYP1A1 activity in less than two minutes. The acetylene group is probably responsible for irreversible enzyme inactivation. Docking simulations revealed the orientations of ethynylflavones in the CYP1A1 binding site with the acetylene group toward the heme. Only 2′-ethynylflavone demonstrated another orientation in the CYP1A1 cavity; this compound appeared to be a selective inhibitor of CYP1A2. In all the studied ethynylflavones docked to CYP1A2, acetylene groups were oriented away from the heme [54].

### 5.5. Stilbenoids

Since the 1990s, natural stilbenoids—*trans*-resveratrol (3,4′,5-trihydroxy-*trans*-stilbene; RESV), pterostilbene (3,5-dimethoxy-4′-hydroxy-*trans*-stilbene), and piceatannol (3,4,4′,5-tetrahydroxy-*trans*-stilbene)—have been extensively studied in relation to chemoprevention [105]. RESV is a natural polyphenol found in grapes, berries, and peanuts, showing well-characterized beneficial bioactivities [106, 107]. It efficiently and selectively inhibits CYP1 activities [108, 109], although its bioavailability in humans was determined as poor [110] due mainly to conjugation reactions with sulphuric acid and glucuronic acid. In the last two decades, natural and synthetic RESV analogues have been studied in the context of their interaction with CYP1s. It has appeared that natural *trans*-resveratrol analogues—pinostilbene, rhapontigenin, desoxyrhapontigenin, and pterostilbene, which possess some of the hydroxyl groups substituted by methoxy groups—are more potent CYP1A1 and CYP1A2 inhibitors than *trans*-resveratrol [111, 112]. Therefore, the substitution of hydroxyl groups with methoxy substituents efficiently influenced the affinity of compounds to active sites of cytochromes and, moreover, improved bioavailability by preventing polyphenol metabolism. Consequently, the interest focused on synthetic derivatives of *trans*-stilbene appeared to be more promising with regard to their interaction with CYP1 enzymes.

The pattern of substituents linked to the *trans*-stilbene core exerts a decisive effect on the affinity of stilbenoids to active sites of cytochromes P450 family 1. The positions of some substituents influence the ligand orientation and interactions with amino acid residues in the enzyme active site, affecting the distance to the heme, which determines the course of enzymatic reaction. In the studies of Chun et al. [75, 112–114], 3,5,2′,4′-tetramethoxy-*trans*-stilbene and 2,4,2′,6′-tetramethoxy-*trans*-stilbene were identified as very potent CYP1B1 inhibitors, indicating a distinctive role of methoxy substituents in positions 2 and 4, as well as 2 and 6 in the inhibition of CYP1B1 activity.

**Figure 5:**
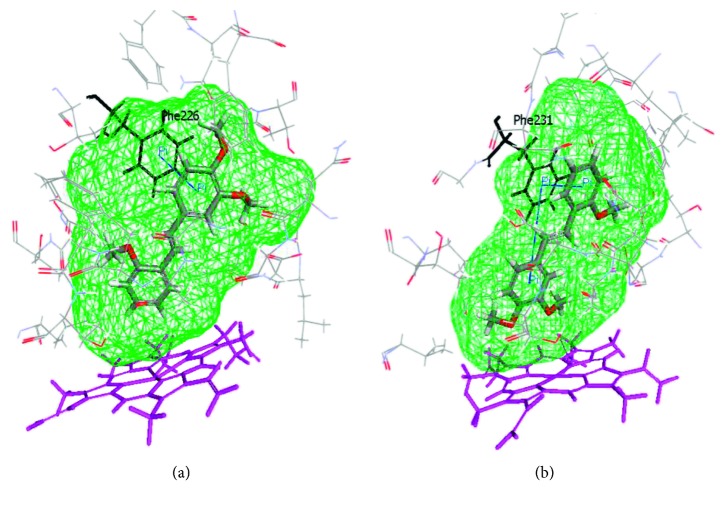
2′,3,4-trimethoxy-*trans*-stilbene docked to the CYP1A2 (a) and CYP1B1 (b) binding sites. Amino acid residues surrounding the active sites are visualized with Phe226 and Phe231 in black colour. The heme is represented as a stick model in pink. The solid blue lines represent *π*-*π* stacking interactions.

The design of the series of polymethoxy-*trans*-stilbenes with the constant motif of 3,4-dimethoxyphenyl influenced the way the ligands were oriented in the enzyme binding site [56]. The molecular docking of *trans*-stilbenes to the CYP1A2 active site showed the most favourable orientation with the ring possessing the altering pattern of substituents directed to the heme (orientation A; Figure 5(a)). However, in CYP1B1, 2′,3,4-trimethoxystilbene was oriented with 3,4-dimethoxyphenyl directed toward the heme (orientation B; Figure 5(b)). This orientation occurred in 17 out of a total of 20 poses and was energetically favourable in comparison to orientation A; the interaction energy and binding energy for the ligand calculated for orientation B was higher by 12 kcal/mol and 40 kcal/mol, respectively [56]. A very strong affinity of 2′,3,4-trimethoxystilbene to the CYP1B1 binding site was expressed by the highest value of binding energy (Δ*G*) in comparison to other compounds of the series. The analysis of the interaction between ligands and amino acid residues in the CYP1B1 active site demonstrated the occurrence of *π*-*π* stacking interactions for both phenyl rings of 2′,3,4-trimethoxystilbene with Phe231 (Figure 5), whereas a hydrogen bond was observed only for the opposite ligand orientation (Figure 5(a)),which was less energetically favourable [56]. In this bonding, Gln332 was engaged; the same amino acid residue formed a hydrogen bond with 4′-methylthiostilbenes. Therefore, it may be supposed that the effect of hydrogen bonds on the ligand affinity to the cytochrome P450 active site is not of primary importance. Hydrophobic interactions between methoxy groups and amino acid residues seem to be more essential in determining the inhibitor affinity to cytochrome P450. Moreover, 2′,3,4-trimethoxy-*trans*-stilbene is characterized by a high selectivity of action; it inhibits CYP1B1 90 times more strongly than CYP1A1 and 830 times more strongly than CYP1A2. Thus, the 2′,3,4-triMS molecule appeared to be a comparably effective CYP1B1 inhibitor than the molecules designed earlier [106–108], demonstrating an effective inhibitory action of the compound with a pattern of methoxy groups in positions 2′, 3, and 4 [56].

In search of novel CYP1 inhibitors, methylthiostilbene derivatives were designed and synthesized [63, 77]. The orientation of a series of polymethoxy-*trans*-stilbene derivatives containing a 4′-methylthio substituent in the CYP1 active sites was studied, and molecular interactions between ligands and amino acid residues of the enzyme pocket were estimated. The orientation with a 4′-methylthiophenyl ring toward the heme for the studied derivatives in CYP1A2 and CYP1B1 active sites was the most favoured one. For this series of compounds, Phe226 and Phe260 in CYP1A2 and Phe231 in CYP1B1 were involved in *π*-*π* stacking interactions that stabilized the orientation of ligands in the enzyme active sites. Additionally, for some of the examined compounds docked to CYP1B1, an active site hydrogen bond was formed with Gln332. However, it should be mentioned that the occurrence of the hydrogen bond did not correlate with the inhibitory effect on enzyme activity. An important role is assigned to the hydrophobic interactions that may have an effect on the closer contact of docked molecules with the Fe atom of the prosthetic group, resulting in the hydroxylation of ligands. For 3,4,5-trimethoxy-4′-MTS and 2,4,5-trimethoxy-4′-MTS, the distances of C atoms in 3′ and 5′ positions to the Fe atom were shorter than 4.5 and 5.5 Å, respectively.

Stilbene derivatives better fit in the CYP1A1 binding site exhibiting a planar long strip cavity than in the CYP1A2 binding site with a more triangular shape [53], for example, the selective CYP1A1 and CYP1B1 inhibitor, 2,3,4-trimethoxy-4′-methylthio-*trans*-stilbene, did not fit the shape of the CYP1A2 binding pocket. The low affinity of 2,3,4,-trimethoxy-4′-methylthio-*trans*-stilbene to the CYP1A2 binding site was additionally confirmed by a high strain energy (103.09 kcal/mol). By comparison, in the CYP1B1 binding site, the strain energy for 2,3,4-trimethoxy-4′-methylthio-*trans*-stilbene was only 40.70 kcal/mol [77].

Another derivative, 2-methoxy-4′-methylthio-*trans*-stilbene, was found to be a selective and potent CYP1A1 inhibitor. Interestingly, its analogue, 2,4′-dimethoxy-*trans*-stilbene, was not so effective. For this derivative, docked to the CYP1A1 binding site, a high number of nonbonded molecular interactions were observed. However, the binding of 2-methoxy-4′-methylthio-*trans*-stilbene was not favourable energetically [63].

## 6. Other Ligands

Most of the ligands of CYP1s are compounds with established pharmacological activity. They include drugs metabolized by the constitutive liver isozyme CYP1A2. A molecular docking of phenacetin and furafylline to CYP1A1 and CYP1A2 active sites was first performed by Lewis and Lake [22] with the use of homology models based on the CYP102 crystal structure. In 2012, Huang et al. studied the isoform-selective metabolism of phenacetin and acetaminophen with the use of the CYP1A2 crystal structure and homology model of CYP1A1 [49].

More recently, the metabolism of drugs selected from the Drug Bank comprising 1,528 drugs approved by the FDA was analyzed with the use of crystal structures of all isozymes of CYP family 1. The substrates were divided into three groups: substrates having a single site of metabolism (SOM) but showing a different preference to get metabolized by CYP1A1, CYP1A2, and CYP1B (e.g., carvedilol, phenacetin, and bufuralol); substrates that are metabolized by any of the three isoforms (e.g., chloroquine and haloperidol); and substrates that show a different SOM and a different preference to isozymes (17-*β*-estradiol) [57]. Differences in substrate specificity among CYPs were studied for melatonin, debrisoquine, theophylline, clozapine, and lidocaine [60]. The regioselectivity of CYP1A2-mediated metabolism was investigated for caffeine, theophylline, acetanilide, naproxen, tacrine, amitriptyline, clozapine, and alkoxyresorufins by Jung and coworkers [67]. More recently, regioselective metabolism of acetaminophen catalyzed by CYP1A2 was proved through a molecular dynamics procedure [80].

Although many reports have demonstrated the inhibitory activity of alkaloids against the activities of CYPs [115], only for caffeine, theophylline [80], and rutaecarpine and its derivatives [29, 116], structural modeling has been performed. A good fitting of rutaecarpine with the binding site of the CYP1A2 model based on the rabbit CYP2C5 as a template was found [29]. Two hydrogen bonds can be formed between the keto and N14 groups of rutaecarpine and Thr208 and Thr473 residues of CYP1A2. The planar molecule of rutaecarpine forms *π*-*π* stacking interaction between the C-ring and aromatic ring of the Phe205 residue. A possible orientation of coumarin in CYP1A1 and CYP1A2 binding sites for 3,4-epoxidation was demonstrated with enzyme structures based on the CYP2A5 crystallographic template. Key amino acid residues—Ser113, Phe205, Tre298, and Phe352—were identified for coumarin docked to CYP1A1 [116]. In the CYP1A2 binding site, Tre113, Phe205, and Tre298 participate in ligand-enzyme interaction. In both enzymes, Phe205 is responsible for *π*-*π* stacking interaction with aromatic rings of the substrates. Both CYP1A1 and CYP1A2 metabolize coumarin in the same molecular positions.

In the context of cancer chemoprevention, naturally occurring isothiocyanates (ITCs) as inhibitors of CYP1s were studied. Sulforaphane, which is one of the most active chemopreventive agents and inhibitors of CYP1A1 activity, was docked to the CYP1A1 active site. Two hydrogen bonds between the nitrogen atom of sulforaphane and the hydrogen of the amino groups of Arg110 were found [51]. Moreover, sulforaphane suppressed the aryl hydrocarbon receptor (AHR) by binding to its ligand binding domain with hydrogen bonds. However, the studies did not explain the lack of potential to reduce the genotoxicity of TCDD.

Emodin is a natural anthraquinone extracted from *Rheum emodi*, a plant used in Chinese medicine. Among the CYPs studied (CYP1A1, CYP1A2, and CYP2B1), this anthraquinone demonstrated the most potent inhibitory activity toward CYP1A2 with the IC_50_ value of 3.73 *µ*M [117]. In the PubChem and ZINC chemical databases, 12 emodin analogues were found for further studies. Two of them (1-amino-4-chloro-2-methylanthracene-9,10-dione (compound 1) and 1-amino-4-hydroxyanthracene-9,10-dione (compound 2)) inhibited CYP1A2 with IC_50_ < 1 *µ*M, but only compound 1 was a mechanism-based inhibitor of both CYP1A1 and CYP1A2. Molecular docking revealed the orientation of molecules in the binding site, which make possible the abstraction of hydrogen from the 2-methyl group present only in compound 1. As a result, a benzylic carbon radical intermediate might be produced, which, after rearrangement, could form an irreversible complex with the enzyme. The radical can react with the iron-bound hydroxyl radical to form a hydroxylated metabolite, which acts as an inactivator of CYPs. A 2-methyl group of compound 1 docked to the CYP1A1 and CYP1A2 binding sites was found close to the heme moieties [117].

Combining *in vitro* studies with a computational approach enabled us to identify compounds that may interact with other drugs. This strategy appeared to be useful in the investigation of drug-drug interactions which are of great clinical importance in relation to multidrug disease treatment. Inhibitory effects of 91 kinase inhibitors (KIs; 80 KIs are not used clinically and 11 are FDA-approved KIs) on human CYPs—CYP1A2, 2C9, 2D6, and 3A4—were determined. For the majority of the KIs under analysis, a differential inhibitory effect on CYP enzymes was observed; fifteen compounds exhibited a potent inhibitory effect on CYP activities (IC_50_ ≤ 1 *µ*M). Clinically used KIs—nilotinib, sunitinib, and imatinib—appeared to be potent CYP1A2 inhibitors with IC_50_ values of 0.92–1.23 *µ*M [79]. In the docking validation studies, 20 compounds among 22 inhibitors selected in high-throughput *in vitro* studies (90.9%) demonstrated a high docking interaction energy. Three functional residues (Phe226, Phe125, and Asp320) in the active site of CYP1A2 were identified [79].

## 7. The Site of Metabolism

Identifying the sites of metabolism (SOMs) can play a decisive role in the design of drugs displaying desirable properties. The basic computational methods used for predicting SOMs and the structures of metabolites are QSAR, 3D QSAR, the pharmacophore-based method, molecular docking, molecular dynamics simulation, and a combined approach which is applied in numerous studies [118, 119]. Computational techniques used in studies of xenobiotic metabolism are classified into the ligand-based approach and the structure-based approach. Taking into account the scope and limitations of these techniques, the combination of both ligand-based and structure-based approaches seems to be promising. In order to predict the site of metabolism, a molecular docking of substrates to the binding sites of cytochromes P450 may be performed. Lewis et al. [116] used CYP1A1 and CYP1A2 homology structures based on the CYP2C5 crystallographic template in a study of coumarin metabolism, finding a good correlation for binding energies determined experimentally and with the use of molecular docking.

In studies of stilbene derivatives, the molecular docking of 4′-methylthio-*trans*-stilbene derivatives to the CYP1A2 binding site confirmed the orientation of the 4′-methylthiophenyl ring of 2,4,5-trimethoxy-4′-methylthio-*trans*-stilbene and 3,4,5-trimethoxy-4′-methylthio-*trans*-stilbene in the close vicinity of the heme, allowing the reaction of hydroxylation at C-3′ to take place [77].

From all the binding modes obtained as a result of the docking procedure, possible metabolic sites of a substrate are assigned to the atoms located within 5 Å from the Fe atom [120]. Molecular docking takes into account binding affinities and steric effects related to the conformation of an active site. The best results of SOM prediction were obtained with the approach combining molecular docking with semiempirical molecular orbital calculations that provide the activation energy characterizing the reactivity of a substrate [67]. Possible binding modes of CYP1A2 substrates were analyzed using automated docking with the use of the crystal structure of CYP1A2. For caffeine and theophylline, the SOMs found were in accordance with experimental data typing N1-CH_3_, N7-CH_3_, and N3-CH_3_ as sites of the formation of primary and secondary metabolites [67].

Biotransformation studies of drugs can be performed with the use of molecular docking and molecular dynamics. Prediction of the formation of toxic metabolites is particularly important [121]. Two acetylcholinesterase inhibitors, derivatives of *p*-aminophenol and succinic anhydride, were tested in order to determine whether toxic metabolites are generated as in the case of *N*-acetyl-*p*-aminophenol (APAP) which is metabolized by CYP1A1 and CYP2B1 to toxic *N*-acetyl-*N*-hydroxy-*p*-aminophenol. Molecular dynamics confirmed that the amide group of APAP interacted with the heme iron of CYP1A1, and as a result of N-oxidation, a toxic intermediate (*N*-acetyl-*p*-benzoquinone imine) was formed. For both studied inhibitors docked to CYP1A1, this kind of interaction was not found. Instead, an aryl hydroxyl hydrogen interaction with the heme was observed. The results obtained in silico correlated well with the studies *in vitro*, which revealed the formation of only hydroxylated metabolites as a result of the metabolism of the studied inhibitors by rat liver microsomes [121]. The regioselectivity of APAP metabolism was studied with molecular docking and molecular dynamics, followed by 2D USP free energy scanning. CYP1A2 and CYP2E1, the enzymes with compact active sites, were found to be major APAP metabolizers [80]. APAP formed more interactions in CYP1A2 and CYP2E1 binding sites as compared with the more voluminous binding sites of CYP3A4 and CYP2C9, which resulted in stabilized binding states and a longer residence time.

## 8. Conclusions

Computational docking studies contribute to a better understanding of ligand-enzyme interactions at a molecular level. Studies with the use of computational procedures provide a rationalization of the selectivity of ligands toward CYP1 isozymes. The elucidation of cytochrome P450 family 1-specific activities at a molecular level is of great importance with regard to novel and potent drug design and drug-drug interactions. Although computational methods have been significantly developed, a further improvement of virtual procedures could have an impact on their usefulness in the design of drugs targeting CYP enzymes by predicting the site of metabolism and drug-drug interactions and determining the potential toxicity of substrates and their metabolites.

Molecular docking helped to visualize spatial ligand fitting and molecular interactions occurring in the enzyme active site. The hypothesis is that not a single substituent but a pattern of substituents determines the shape of a molecule and influences a ligand's affinity to a binding site. The pattern of substituents exerts an effect on the ligand orientation in the enzyme active site, which in the case of some ligands is stabilized by hydrophobic interactions, especially *π*-*π* stacking interactions. In the case of ligands that are substrates of enzymatic reactions, the distance between a ligand and the prosthetic group is essential for the course of reaction. Combining experimental studies on enzymatic reactions with a computer analysis of ligand-active site interactions are expected to produce valuable results, useful in the design of molecules with a desired activity.

Summarizing the achievements of the reports reviewed, many authors emphasize the versatility and plasticity of CYPs. In silico methods in the studies of ligand-CYP isozyme interactions provide a predictive model based mainly on van der Waals interactions, whereas electrostatic interactions do not play a considerable role here. The ligand can change its conformation through adaptation to the shape of the enzyme active site. Analyses of the ligand shape revealed the essential role of shape complementarity to the cavity of the enzyme binding site. Amino acid residues and water molecules can form hydrogen bonds that stabilize the ligand-enzyme complex.

Computational structure-based ligand design is a promising technique which enables an efficient analysis of preclinical drug candidates. Docking may be used to provide information about the conformation of a bioactive ligand and its position in the binding site. Knowing the orientation of a ligand helps to predict the site of metabolism.

## Figures and Tables

**Table 1:** The summary of studies on CYP1-ligand interactions.

Cytochrome P450	Ligand	Methods	Notes	References
CYP1A1 and other cytochromes				
CYP1A1 and CYP1A2	Aromatic amines, heterocyclic amines, aromatic hydrocarbons (benzo(*a*)pyrenemethylcholantrene), phenacetin, furafylline, and 7-methoxyresorufin	Homology modeling based on the CYP102 crystal structure	Human, mouse, rabbit, and trout CYP sequences	[22]
CYP1A1	7-Ethoxyresorufin, 7-methoxyresorufin, and benzo[*a*]pyrene	Homology modeling		[28]
CYP1A1, CYP1A2, and CYP1B1	Rutaecarpine and its derivatives	Homology modeling		[29]
CYP1A1	Arachidonic acid and eicosopentaenoic acid	Homology modeling	Molecular docking explains regiospecificity of metabolism	[30]
CYP1A1 and CYP1A2	7-Methoxyresorufin and 7-ethoxyresorufin	Homology modeling	Active site mutations in human CYP1A1 and CYP1A2	[31]
CYP1A1 and CYP1A2	Dietary flavonoids	Homology modeling		[32]
CYP1A1	B[*a*]P, B[*a*]P-7*R*, 8*R*-dihydrodiol, B[*a*]P-7*S*, 8*S*-dihydrodiol, eicosapentaenoate, and arachidonate	Homology modeling	Regioselectivity	[33]
CYP1A1	Ethoxyresorufin	Homology modeling		[34]
CYP1A1	B[*a*]P	Wild-type and exon 6 del CYP1A1 homology models		[35]
CYP1A1, CYP1A2, and CYP1B1	Alkoxyl derivatives of 7,8-dehydrorutaecarpine	Homology models based on the crystal structure of rabbit CYP2C5		[36]
CYP1A1	B[*a*]P, TCB, and TCDD	Rat, human, scup, and killifish homology models		[37]
CYP1A1	Representative ligands: *α*-naphthoflavone and benzothiazole	Homology modeling		[38]
CYP1A1 and CYP1A2 (CYP2A6 and CYP2B1)	Arylacetylenes	CYP1A2 crystal structure (PDB: 2HI4) and homology model of CYP1A1	Distances of ligands to heme, Fe, and Phe residues were analyzed	[39]
CYP1A1	Benzoxazoles and benzothiazoles	CoMFA, homology modeling, and molecular docking		[40]
CYP1A1, CYP1A2, and CYP1B1 (CYP2C9 and CYP3A4)	33 flavonoid derivatives	PDB: 2HI4 and homology models of CYP1A1 and CYP1B1	Hydroxyl and methoxy derivatives of flavone more potent as CYP inhibitors	[41]
CYP1A1, CYP1A2, and CYP1B1	Methoxyflavonoids	PDB: 2HI4 and homology models of CYP1A1 and CYP1B1	Important amino acid residues	[42]
CYP1A1 and CYP2B1	*p*-Aminophenol-succinic acid derivatives (acetylcholinesterase inhibitors)	Homology modeling of rat CYPs based on structures of CYP1A2 and CYP3A4 and molecular dynamics	Biological experiments on rat microsomes induced with 5,6-benzoflavone and phenobarbital	[43]
CYP1A1, CYP1A2, and CYP1B1	17-*β*-Estradiol	PDB: 2HI4 and homology models of CYP1A1 and CYP1B1	Important amino acid residues	[44]
CYP1A1	3,3′,4,4′,5-Pentachlorobiphenyl	Homology modeling	Rat and human recombinant microsomes	[45]
CYP1A1 and CYP1B1	Resveratrol and its derivatives	Homology modeling based on CYP1A2 crystal structure		[46]
CYP1A1 and CYP1B1	Dietary flavonoids	Homology models based on the structure of CYP1A2 (PDB: 2HI4)		[47]
CYP1A1 and CYP1A2 (CYP1A6 and CYP2B1)	Flavone propargyl ethers	CYP1A2 crystal structure (PDB: 2HI4) and homology model of CYP1A1	Flavone propargyl ethers are more potent inhibitors of CYP1A1 and CYP1A2 than the parent hydroxy flavones	[48]
CYP1A1 and CYP1A2	Phenacetin and acetaminophen	CYP1A2 crystal structure (PDB: 2HI4) and homology model of CYP1A1	Isoform-selective metabolism	[49]
CYP1A1 and CYP1B1	Polycyclic aromatic hydrocarbons	Homology modeling		[50]
CYP1A1	Sulforaphane	The tertiary structure of CYP1A1 was generated with the combination methods of threading, ab initio modeling, and structural refinement	Sulforaphane failed to reduce the genotoxic effect of TCDD in yeast cells	[51]
CYP1A1	Pyrimidobenzothiazole (NSC745689)	Homology modeling and molecular dynamics		[52]
CYP1A1, CYP1A2, and CYP1B1 (CYP2A6 and CYP2B1)	Pyranoflavones		Molecular surface images generated from UCSF Chimera	[53]
CYP1A1 and CYP1A2	Ethynylflavones	PDB: 4I8V and PDB: 2HI4	Selective inhibitory activity toward CYP1A1	[54]
CYP1A1	Polychlorinated dibenzo-*p*-dioxins and coplanar polychlorinated biphenyls	Homology modeling	Rat and human CYP1A1	[55]
CYP1A1, CYP1A2, and CYP1B1	Polymethoxystilbenes	PDB: 4I8V, PDB: 2HI4, and PDB: 3PM0	Potent and selective inhibitory activity of 2,3′,4′-trimethoxy-*trans*-stilbene	[56]
CYP1A1, CYP1A2, and CYP1B1	30 drugs metabolized by CYPs	PDB: 4I8V, PDB: 2HI4, and PDB: 3PM0	MetaSite	[57]
CYP1A1 and CYP1A2	22 aromatic hydrocarbons and 3 fluorogenic alkoxyaryl compounds	PDB: 4I8V and PDB: 2HI4	CYP1A variants	[58]
CYP1A1, CYP1A2, and CYP1B1	Alkoxyresorufins	Homology modeling	Baikal seal and human CYPs	[59]
CYP1A1, CYP1A2, and CYP1B1	5F-203, 5-aminoflavone, 17-*β*-estradiol, melatonin, debrisoquine, theophylline, clozapine, and lidocaine	PDB: 4I8V, PDB: 2HI4, and PDB: 3PM0	Differences in substrate specificity among CYPs	[60]
CYP1A1	Naringenin and dihydroxybergamottin	Rat homology model, human PDB: 4I8V, and molecular dynamics		[61]
CYP1A1	Compounds selected by virtual screening of databases	Database screening, Hypo1; metabolite prediction study, MetaSite software; molecular docking studies; and molecular dynamics simulations	Antiproliferative activity on MDA-MB-435 human cells and two lead compounds with antitumor activity against MDA-MB-435 line	[62]
CYP1A1, CYP1A2, and CYP1B1	Polymethoxy- and methylthio-*trans*-stilbene derivatives	PDB: 4I8V, PDB: 2HI4, and PDB: 3PM0		[63]

CYP1A2 and other cytochromes				
CYP1A2	Caffeine and MeIQ	Homology model based on CYP BM3 crystal structure		[64]
CYP1A2 (CYP2D6 and CYP3A4)	Selected substrates	Homology modeling	Substrate selectivity studies	[65]
CYP1A2	7-Methoxyresorufin	Homology model based on the crystal structure of CYP2C5	Hydrogen bonds and *π*-*π* stacking with Phe226	[66]
CYP1A2 (CYP2A6, CYP2C9, CYP3A4, and CYP2E1)	Caffeine, theophylline, acetanilide, phenacetin, 7-methoxycoumarin, 7-ethoxycoumarin, 3-cyano-7′-ethoxycoumarin, naproxen, tacrine, amitriptyline, clozapine, and 7-ethoxyresorufin	PDB: 2HI4	Regioselectivity prediction of CYP1A2-mediated metabolism	[67]
CYP1A2	Methoxyresorufin and ethoxyresorufin	CYP1A2 homology model and crystal structure PDB: 2HI4 and homology structures of CYP1A2 mutants		[68]
CYP1A2	Virtual screening of CYP1A2 ligands	PDB: 2HI4 and automated docking (Gold version 3.2)	Prediction of the site of metabolism	[69]
CYP1A2	Structurally diverse CYP1A2 ligands (substrates and inhibitors)	PDB: 2HI4 and molecular dynamics	Versatility and plasticity of the CYP1A2 active site	[70]
CYP1A2 (CYP2C9)	Chrysin, 7,8-benzoflavone, 7-hydroxyflavone, and warfarin	PDB: 2HI4 and molecular dynamics		[71]
CYP1A2	Phenacetin	PDB: 2HI4	Wild-type and mutant forms of enzyme	[72]
CYP1A2	Virtual screening of 971 herb compounds	Pharmacophore searching and docking procedure to CYP1A2 crystal structure (PDB: 2HI4)	Herb-drug interactions	[73]
CYP1A2 (CYP2A6, CYP2C9, and CYP2D6)		PDB: 2HI4 and molecular dynamics	Flexibility at normal and high-pressure conditions (300 MPa)	[74]
CYP1A2 and CYP1B1	Polymethoxy-*trans*-stilbenes	PDB: 2HI4 and homology model of CYP1B1	Potent and selective inhibitory activity of 2,4,2′,6′-tetramethoxy-*trans*-stilbene	[75]
CYP1A2	7,8-Benzoflavone, oroxylin, and wogonin	PDB: 2HI4, binding free energy analysis with the MM-PBSL method, and molecular dynamics		[76]
CYP1A2 and CYP1B1	4′-Methylthio-*trans*-stilbene derivatives	PDB: 2HI4 and PDB: 3PM0		[77]
CYP1A2	7-Ethoxyresorufin	PDB: 2HI4, ensemble docking, and molecular dynamics	Phe186Leu mutation	[78]
CYP1A2 (CYP2C9, CYP2D6, and CYP3A4)	Kinase inhibitors	PDB: 2HI4	Drug-drug interactions	[79]
CYP1A2 (CYP2A6, CYP2C9, CYP3A4, and CYP2E1)	Acetaminophen	Large-scale 2D umbrella sampling, PDB: 2HI4, and molecular dynamics	Regioselectivity	[80]
CYP1A2		The initial structure of wild-type CYP1A2 (CYP1A2.1) constructed from the CYP1A2 crystal structure PDB: 2HI4, and CYP1A2 mutants constructed from CYP1A2.1 refined after molecular dynamics simulation	Influence of amino acid mutations on the 3D structure and dynamic properties of the enzyme	[81]

CYP1B1				
CYP1B1	17-*β*-Estradiol, *α*-naphthoflavone, 7-ethoxycoumarin, 7-ethoxyresorufin, bufuralol, and benzo(*a*)pyrene-7,8-diol	Homology model based on the structure of CYP2C5	Allelic variant effects on metabolism	[82]
CYP1B1	17-*β*-Estradiol	Molecular dynamics simulations of homology-modeled structures	PCG-associated mutants	[83]
CYP1B1	7,8-Benzoflavone derivatives	PDB: 3PM0; MOE docking program	Inhibitors that eliminate CYP1B1-mediated drug resistance	[16]

B[*a*]P: benzo[*a*]pyrene; TCB: 2,3′,4,4′-tetrachlorobiphenyl; TCDD: tetrachlorodibenzo-*p*-dioxin; PCG: primary congenital glaucoma.
